# Generic medical concept embedding and time decay for diverse patient outcome prediction tasks

**DOI:** 10.1016/j.isci.2022.104880

**Published:** 2022-08-04

**Authors:** Yupeng Li, Wei Dong, Boshu Ru, Adam Black, Xinyuan Zhang, Yuanfang Guan

**Affiliations:** 1 Merck & Co., Inc., Rahway, NJ, USA; 2 Ann Arbor Algorithms Inc., Ann Arbor, MI 48104, USA; 3 Odysseus Data Services, Cambridge, MA 02142, USA; 4 Department of Computational Medicine and Bioinformatics, University of Michigan, Ann Arbor, MI 48109, USA

**Keywords:** Health sciences, Bioinformatics, Medical informatics, Automation in bioinformatics

## Abstract

Many fields, including Natural Language Processing (NLP), have recently witnessed the benefit of pre-training with large generic datasets to improve the accuracy of prediction tasks. However, there exist key differences between the longitudinal healthcare data (*e.g.*, claims) and NLP tasks, which make the direct application of NLP pre-training methods to healthcare data inappropriate. In this article, we developed a pre-training scheme for longitudinal healthcare data that leverages the pairing of medical history and a future event. We then conducted systematic evaluations of various methods on ten patient-level prediction tasks encompassing adverse events, misdiagnosis, disease risks, and readmission. In addition to substantially reducing model size, our results show that a universal medical concept embedding pretrained with generic big data as well as carefully designed time decay modeling improves the accuracy of different downstream prediction tasks.

## Highlights

• This work develops a pre-training scheme for longitudinal healthcare data
• The method leverages the pairing of medical history and a future event
• We created a universal medical concept embedding pretrained with generic data
• We designed a time-decay method for medical concept data

## Graphical abstract

**Figure:**
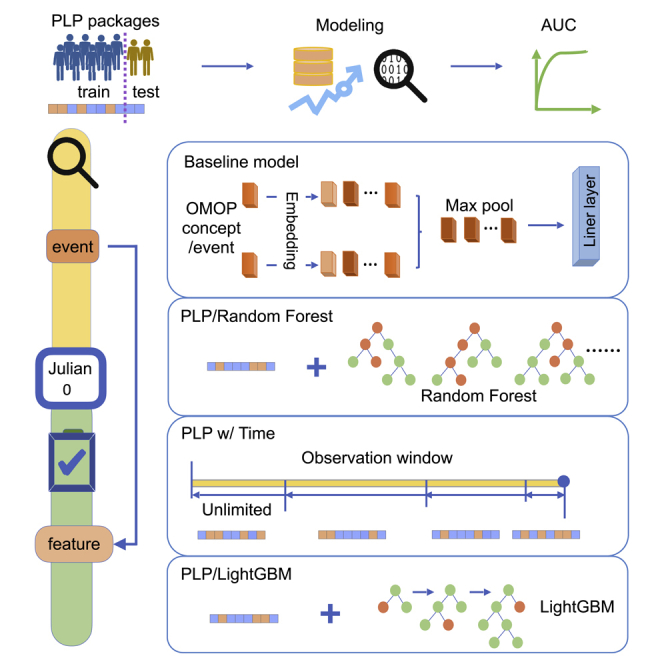

## Introduction

Longitudinal healthcare data such as medical claims from millions of patients are routinely collected and are enriched with structured or unstructured data elements, *e.g.*, diagnosis, medication, procedure, demographics, lab tests, medical images, and clinical notes. This information can be used to predict diverse health outcomes such as survival, drug treatment effects, and so forth. Effective utilization of such data remains a challenge that demands the integration of optimal machine learning models, a widely adopted data format standard, and software that is capable of efficiently handling massive datasets.

Neural network models, specifically those based on transformers (Vaswani et al., 2017), have recently revolutionized the natural language processing (NLP) field. BERT (Devlin et al., 2018), when it was published, achieved state-of-the-art performance on multiple NLP tasks, and many further improvements have since been published (Lan et al., 2019; Liu et al., 2019; Yang et al., 2019). It is widely accepted that a neural network model pretrained on a large generic dataset can benefit specific downstream NLP applications, especially those with limited training data. The outcome of such pre-training is the embedding of tokens from a dictionary that is shared by all tasks of a certain domain. There is a close resemblance between NLP data (sequences of words from a dictionary) and longitudinal healthcare data (sequences of events that can be represented by tokens from standard medical concept dictionaries such as ICD10 diagnosis codes and CPT4 procedure codes). Such similarity has inspired the applications of models originated in NLP to longitudinal healthcare data and have shown promising results (Choi et al., 2016; Beam et al., 2019; Li et al., 2020; Lahlou et al., 2021; Rasmy et al., 2021; Si et al., 2021; Zeng et al., 2021). Nevertheless, the superiority of the transformer-based approach in longitudinal healthcare data cannot yet be established because of the following two reasons.

First, previous studies have usually assumed the effectiveness of certain neural-network-based models, and have not conducted a systematic comparison against some very successful tree-based methods such as LightGBM (Ke et al., 2017), which are still dominating many machine learning benchmarks. There have not been any side-by-side comparisons between neural network models and tree-based models in longitudinal healthcare data. The breakthrough of BERT in NLP is definitive because some of the benchmarks that BERT tops are standard benchmarks that have been used in the NLP community long before the deep-learning breakthrough. The longitudinal healthcare data analytic community does not yet have the leverage of such standard benchmarks.

Second, network architectures have become very deep and complicated in NLP applications. Models such as BERT usually involve many hyper-parameters that are very time-consuming to tune. Such complicated software packages are usually used as it is for longitudinal healthcare data and parameters are inherited without scrutiny. Critical differences between NLP and longitudinal healthcare data have so far been neglected.

Recent years have also seen promising progress in longitudinal healthcare data standardization. The OMOP Common Data Model (CDM) was developed to allow systematic analysis of disparate observational databases. The maintaining organization OHDSI and the surrounding community have developed a plethora of useful software for databases conforming to the standard CDM format (OHDSI, 2019). The adoption of the CDM standard provides an opportunity to train general machine learning models across multiple data sources which represent different patient populations, coding systems, and so forth, and to easily apply these models back to new data sources. In addition to data format standardization, a significant step forward made by CDM is the standardization of medical concept vocabulary. An umbrella dictionary is curated that covers ICD-10, RxNorm, SMODED, and more than a hundred other domain-specific vocabularies.

Patient Level Prediction (PLP) (Reps et al., 2018) is an OHDSI software package that is particularly relevant to this study. PLP provides a standard framework to define patient outcome prediction tasks with binary labels. Many open-source studies have been created using the PLP framework. A current limitation of the PLP package is that it is designed to handle individual observational clinical studies which do not usually involve too large a cohort size. It cannot be easily extended to integrate a pre-training step that involves a massive dataset.

In this article, we examined and leveraged the differences between the nature of NLP and longitudinal healthcare tasks and proposed a novel pre-training task based on binary classification between a true future event and a randomly sampled control event. Second, we explored the effectiveness of pre-training with large longitudinal healthcare datasets and time-decay encoding in improving the model accuracy of downstream-specific prediction tasks. However, such improvements can only be obtained with careful fine-tuning. Third, we conducted a systematic end-to-end evaluation of representative machine learning methods on a collection of PLP studies totaling 10 prediction tasks. We show improved performance of neural networks than current state-of-the-field tree algorithms. But our result does not support the superiority of more complicated architectures, *e.g.* LSTM or transformers, over simpler architectures. This is likely owing to the important difference between NLP tasks where the choices of words in a context are often limited and longitudinal healthcare tasks, where different events may be combined without a strong contextual relationship. Fourth, we implemented a framework for efficient preprocessing of massive longitudinal healthcare data stored in a standard CDM database containing administrative health insurance claims for approximately 69 million unique members using the map-reduce paradigm. The framework efficiently augments, shuffles, and streams the processed samples in mini-batches into mainstream deep-learning frameworks such as PyTorch for model training. The software is made open source to the community.

## Results

### Baseline deep learning model on longitudinal healthcare data performs well compared to the state-of-the-field algorithms

**Figure 1:**
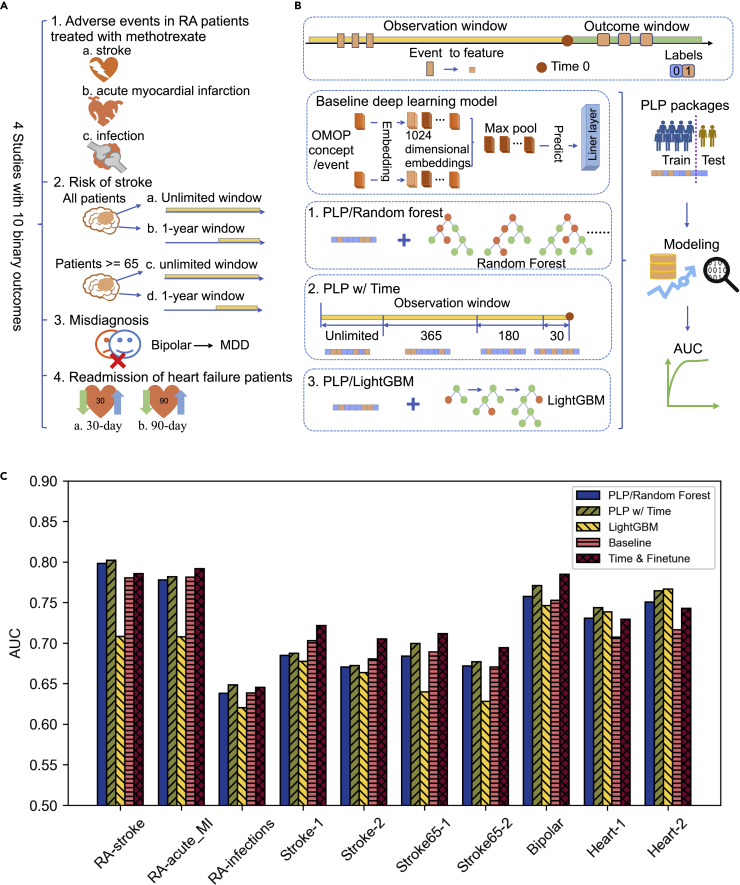
Comparison of baseline neural network models and state-of-the-field traditional machine learning models in predicting different outcomes (A) Selection of 10 cohorts covering adverse health outcomes, disease risk, misdiagnosis, readmission. (B) Baseline neural network architecture, PLP-related tree-based algorithms, and their variations. (C) Performance comparison across baseline methods.

In order to evaluate the generalizability of the algorithms developed in this study, we collected a benchmark of 10 binary patient outcome prediction tasks from 4 studies (Figure 1A). The first study, supported by The European Health Data & Evidence Network (EHDEN) and OHDSI, focused on predicting the risk of adverse health outcomes including stroke, acute myocardial infarction (acute_MI), and infections in patients with adult RA after initiating first-line treatment of methotrexate monotherapy. The second study aimed at predicting stroke risk. The study defines two target cohorts: all patients (Stroke) and patients of age ≥ 65 (Stroke65). For each target cohort, two population settings are used: unlimited risk window (Stroke-1) and risk window of 365 days (Stroke-2). Thus, there are four tasks in total. The third study was to detect the possible misdiagnosis of bipolar disorder as MDD. These three studies are all from previous OHDSI network studies. PLP packages, including the cohort definition, feature extraction, and prediction models, have already been built. We used the published PLP packages to retrieve the patient populations and outcome labels. Feature extraction and prediction models were customized in this work for a fair comparison. The fourth study came from Wang et al. to predict 30-day (Heart-1) and 90-day (Heart-2) readmissions in hospitalized patients with heart failure with HFrEF.

We evaluated different methods using the same training/testing splitting protocol defined by the PLP package (Reps et al., 2018). Briefly, the entire time period is separated into the observation window before the cutoff time, namely time 0, where we extract predictive features, and the outcome window after time 0 (Figure 1B). If an event happens in the outcome window, it is labeled as 1, and otherwise zero. The patients in each cohort are separated into the training set and test set defined by the PLP package for a fair comparison, where the training set features derived from the observation window and the outcome are available for training, and the test set features from the observation window are used as the test. Because all tasks are binary predictions, we used the area under the ROC curve or AUC for the accuracy measurement.

We first compared a baseline deep learning model against three other existing methods (Figure 1C). The baseline neural network model contains an embedding layer, which converts each occurrence of an event, represented as an OMOP concept, into a 1024 dimensional embedding. Then, we took the maximal value of each position of the 1024 dimensional embedding (max-pool). Finally, we added a linear layer to predict the outcome. This baseline model was not pretrained and was without time information. The improved deep learning method (Time & fine-tune) builds on top of the fourth method, but adds in time information and pre-training, which is explained and evaluated in more detail in the following sections.

The first reference method (PLP/Random Forest) uses a random forest as the base learner. In this method, the length of the feature vector is the size of the dictionary of OMOP concepts. A feature is 1 if the vocabulary exists in the observation window, and 0 otherwise. The second method (PLP w/Time) separates the observation time into 30, 180, 365 days, and unlimited before observation cutoff time 0. If a concept occurs within the time period, the corresponding feature is 1, and otherwise 0. Thus, the second method has feature vectors four times as long as the first method. The third method (LightGBM) builds on top of the first method, but changes the base learner to lightGBM.

We found that random forest in general works better than LightGBM, and adding time information consistently improves the accuracy of the random forest method (Figure 1C). Additionally, neural networks are competitive in patient outcome prediction tasks with longitudinal healthcare data. It outperforms tree-based methods in 6 out of 10 cases and thus could serve as our baseline for future development (all performances are included in Tables S2–S5).

### Comparison of the performance of different neural network architectures

**Figure 2:**
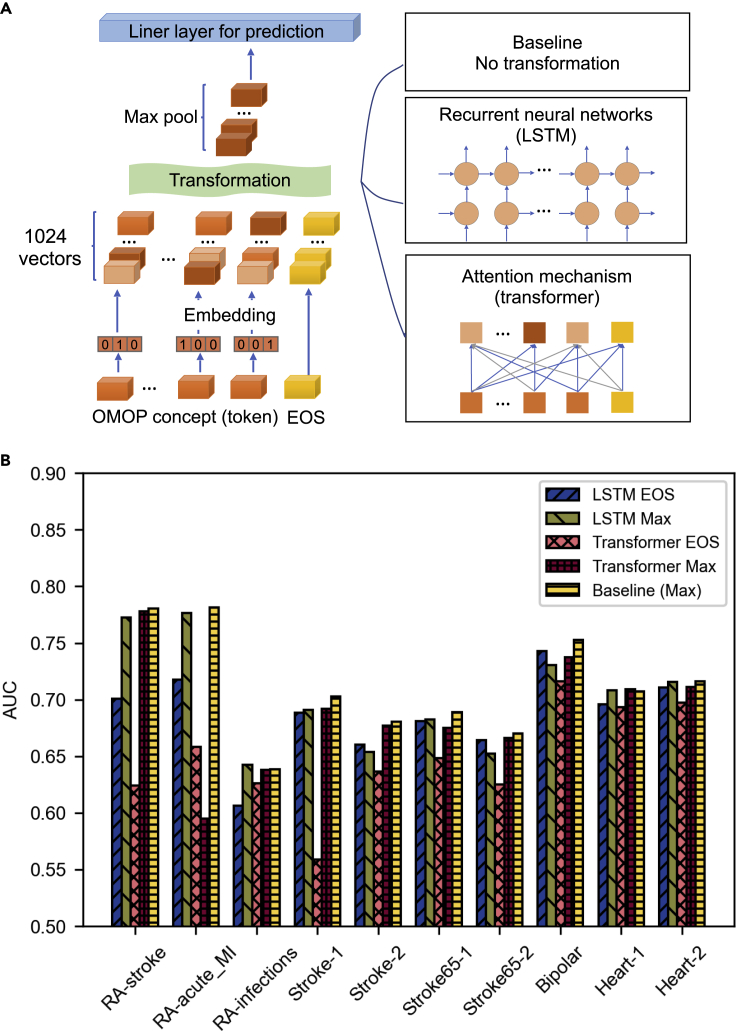
Comparison of alternative neural network architectures (A) We first embedded the one-hot encoded vectors of the concept tokens into 1024 dimensional embeddings. These vectors either go through no transformation (max pool), LSTM, or a transformer. Then we take the max pool of the output to feed in a linear layer for predictions. (B) Performance in AUC for alternative neural network architectures.

We compared the performance of three different neural network architectures. The three architectures share the feature embedding part. We used the concept tokens and their sequential order. The format of data resembles NLP problems (Figure 2A): a sequence of tokens, where each token is the concept ID of a specific event. Each token can be considered as a one-hot-encoded sparse vector with the dimension of the dictionary/vocabulary size (1000s to 100000s; we used 10,000 in the experiments, Figure S1). Because the dictionary is typically big, the input tokens always go through an embedding layer which converts each token into dense feature vectors of relatively low dimensions (1024 in our case). The first network we evaluated, namely, the baseline network, takes the max-pooling of this feature vector as the prediction output. That is, for each position of the 1024 positions, we took the maximal value across all tokens for a particular example. The second and third networks represent two broad categories of neural network architectures that function to extract features or transformation from sequences of low-level feature vectors: recurrent neural networks (RNN), specifically we used LSTM (Hochreiter and Schmidhuber, 1997), and attention mechanisms as represented by Transformers (Vaswani et al., 2017).

For problems with variable sequence lengths, the final sequential layer has to be converted into a time-independent feature vector of fixed dimensionality before it can be fed into the prediction head. There are also two representative methods for this: pooling and using the End-of-Sentence (EOS) element. The EOS token is an artificial token added to the end of each sample specifically to extract information for making predictions. We evaluated multiple pooling methods and found that max-pooling works best. For the baseline architecture, because there is no intermediate feature extraction process for the EOS token to pick up information from other tokens, EOS is not suited for making predictions, but adding the EOS token is still necessary for the model to handle patients with no previous observations at prediction time. After the pooling or EOS feature extraction, all three methods used a fully connected layer in the last step to generate predictions.

Surprisingly, despite their added complexity and better fitting power, neither LSTM nor transformers outperform the baseline architecture (Figures 2B and S2). We also tested a large set of variations of the transformer model differing by depth. This contradicts well-accepted observations that deeper architectures improve accuracy in many other fields such as image processing and NLP. We believe this is owing to the special nature of longitudinal healthcare data, which is relatively flat and lacks a complex structure, and will further discuss this in the next section. We also observe that max-pooling works better for most of the cases. On average, the difference in AUC between max pooling and EOS is 0.016 (2.3%) for LSTM and 0.040 (6.5%) for transformers. In an extreme case, RA-stroke, the gap is as large as 0.072 (10.3%) and 0.15 (24.7%).

### Simulating the effect of time decay improves deep learning model performance

**Figure 3:**
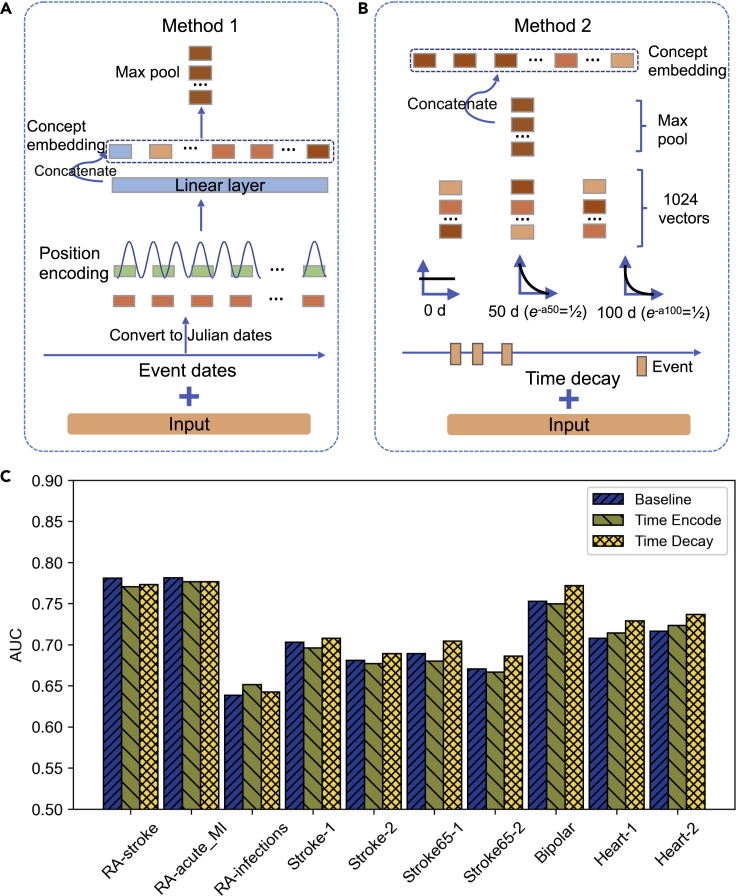
Comparison of performance for different time decay methods added to the baseline model (A) “Time Encode”: using absolute event date to encode the dates. (B) “Time Decay”: using exponential decay to model the reduction of weight over time. (C) Comparison of performance of the two-time decay modeling methods.

We further leveraged time information to improve the model performance. Presumably, the further away an event happened, the less effect this event would have on the outcome. The baseline architecture does not make use of timestamps associated with the events and considered an early event contributed the same as a more recent event. We developed the following two methods to leverage the time information. The “Time Encode” method modifies the positional encoding technique used in BERT (Devlin et al., 2018) to handle time information. We converted the event dates into the number of days as a fixed starting date (Nov 23, 4714 BC, the number of days, which is called Julian days), and used the number of days in the position of the serial number used in BERT’s positional encoding layer. Because the positional encoding layer uses periodic functions such as sin/cos functions, the choice of the starting date does not affect model performance. The high-dimensional time encoding then goes through a linear layer for dimension reduction/feature extraction and is then concatenated to the concept embedding before passing into max pooling (Figure 3A).

In the “Time Decay” method (Figure 3B), we applied exponential time decay before passing the embedding of the events to max pooling. The rationale is that events that occurred at a longer time interval before the cutoff date should have less influence on the prediction results. Specifically, each event during the observational table is associated with a time relevant to the cutoff date. The features extracted from the embedding layer (the 1024 dimensional embedding) for a specific event are down-weighted by an exponential function:

(Equation 1) $$f^{\prime }\left(event\right)=f\left(event\right)\times e^{-at}$$

How fast the influence decays depends on the nature of the events, so we applied three different decays for Equation 1, namely no decay (*a* = 0, and thus *f’* = *f*), 50 days (*a* = (*ln*2)/50), thus the feature value at 50 days is reduced to half, and 100 days (*a*=(*ln*2)/100, and thus the feature value at 100 days are reduced to half). After the transformation of the feature vectors, we took the max pooling of each decaying scheme and concatenated the three decayed versions of embedding, resulting in 3072 dimensional embeddings. We observed that “Time Decay” is better than the baseline method without time information in most cases, except for the two tasks of RA-stroke (Figure 3C). Absolute time encoding alone, however, performed worse than the baseline in more than half the tasks.

### Pre-training the concept embedding with large data repositories improves model performance and reduces model complexity

There is a fundamental difference between longitudinal healthcare data and classical NLP that must be considered in designing the pre-training procedure. The standard pre-training task in NLP is a masked language model, in which a fraction of the words in a sentence are masked out as the prediction tasks. Therefore, in NLP, the choice to fill a missing word in a sentence is usually limited. For example, in the sentence “I ____ happy.” There is a limited set of words (“am,” “was,” “feel,” “felt” …) that can be filled in. In longitudinal healthcare data, however, the relationship between observed events is more of a causal or correlation relationship, and the order of the events is much more flexible. If we mask out an event in a patient’s history, there are many ways to fill in the blank without making the history look unnatural. Thus, the mask-out approach developed in NLP is not suitable for longitudinal healthcare data.

**Figure 4:**
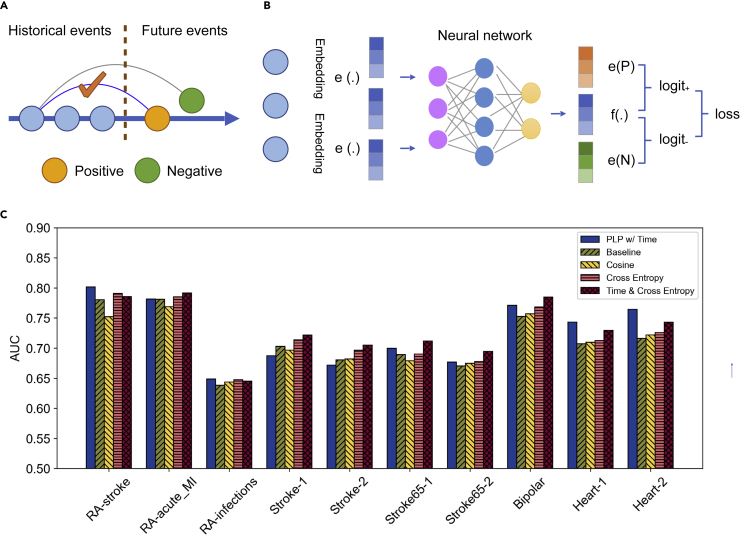
Performance with pre-training where the same baseline architecture was used for pretraining (A) During pre-training, a positive example is created by a pair of concepts that happened to an individual, respectively, during the observation window and outcome window. An example is created by a pair where one concept appeared during the observation and another random concept (not in the outcome window). (B) The embedding of the concept is created by training a neural network using the above positive and negative examples. (C) Performance comparison of different pre-training strategies. Cross-entropy works better than cosine similarity; combining cross-entropy and time decay further improves accuracy.

In order to design a proper pre-training task, one must rely on an assumption that can be generally regarded as true. The assumption is that given any history of observed events, some future events are more likely to occur than others (Figures 4A and 4B). Thus, the model learning goal is to correctly distinguish a real future event versus an event selected at random. In this case, both the history and the future event will be represented as vectors of the same number of dimensions, so they can be properly correlated. Specifically, in a pre-training task, we are presented with a sequence *S* of medical events ordered by timestamps. We considered *Si*, the prefix of the first *i* events, as visible history, with the rest as held-out data. We assume that an event *Pi* (positive) in the held-out data is more likely to occur in real life than an event *Ni* (negative) with a concept randomly sampled from the vocabulary. In other words, *Pi* should be more compatible with the history of *Si* than *Ni*. For the compatibility test, we note that both *Pi* and *Ni* have the embeddings, denoted by *e*(.), and *Si* is converted to a feature vector by the neural network, denoted by *f*(.) We assume the baseline architecture here, *f*(*Si*) is the max pooling of *e*(*C*) for any *C* in *Si*. In general, *f*(.) can be the output of any predictive network architecture with transformers, and so forth. We simply have to make *f*(.) to have the same dimensions as *e*(.), so that concept embedding can be matched against the output of the neural network. The likelihood of the historical events causing future events can be calculated by the dot product of *f*(.), the neural network output of the history, and *e*(.) the embedding of the future event. We follow the common practice of using cross-entropy for binary classification and use the following loss function:

(Equation 2) $$logit_{+}\;=dot\left[e\left(Pi\right),\;f\left(Si\right)\right]$$

(Equation 3) $$logit_{-}=dot\left[e\left(Ni\right),\;f\left(Si\right)\right]$$

Equation 4 $$Pr_{+},\;Pr_{-}=\;softmax\left(logit_{+},\;logit_{-}\right)$$

(Equation 5) $$Li\;=\;-\left[\;log\left(Pr_{+}\right)\;+\;log\left(1-Pr_{-}\right)\right]\;$$

(Equation 6) $$Loss\;=\;sum_{i=1}^{n-1}Li$$

In an architecture with cross-entropy loss, the output of the top linear predictive layer is called the logits. For an N-class classification problem, there are N logits with arbitrary ranges. In our case, we have two classes and we denote the logits by *logit+* and *logit-*. These logits are fed into a softmax layer to generate probability-like scores *Pr*_*+*_ and *Pr*_*-*_ such that the two scores add up to 1. In our case the ground-truth label is always 1 for *P*_*i*_ and 0 for *N*_*i*_, so labels do not appear in Equation 5 for cross-entropy calculation. Note that Eq5 is the sum of the cross-entropy of a pair of positive and negative examples, rather than a single cross-entropy value.

We make one prediction on each prefix (except for the whole sequence itself, for which we cannot sample a held-out event) of the sample and add up the loss function. Using such dense predictions allows us to generate more gradients from each sample and make training converge faster.

For comparison, we evaluated an alternative loss function based on cosine similarity instead of cross-entropy.

(Equation 7) $$Li^{\prime }\;=\;-cos\left[e\left(Pi\right),\;f\left(Si\right)\right]\;+\;cos\left[e\left(Pi\right),\;f\left(Si\right)\right]\;$$

(Equation 8) $$where\;cos\left(a,\;b\right)\;=\frac{dot\left(a,\;b\right)}{\left|a\right|\;\left|b\right|}\;$$

A practical advantage of this pre-training task formulation is that it requires much fewer model parameters than a pre-training task that does multiple-category prediction. For example, with an input of dimensions *D*_*hidden*_ = 1000 and a dictionary of size *D*_*dict*_ = 10,000, the size of the linear prediction head to make a 1-out-of-N prediction is *D*_*hidden*_ *x D*_*dict*_ = 10,000,000. That number can easily exceed the total number of parameters of the rest of the neural network. Such a large linear layer can easily cause the network to overfit.

We observed that cross-entropy is universally better than cosine similarity and adding fine-tune always improves the accuracy of the baseline model (Figures 4C and S3), although sometimes the improvement is small. The figure also shows adding time (with the decay method) further improves accuracy.

## Discussion

We demonstrated in this evaluation a pre-training strategy and time-decay modeling that build competitive neural network models for longitudinal healthcare data. Our results argue against the widely accepted notion that when training data is abundant, going deeper always means higher accuracy. We started our experiments with networks much deeper than presented in this article and found it very difficult to achieve the level of accuracy that can be easily obtained by trees. We showed that more advanced architectures, like LSTM and transformers, are inferior to the seemingly trivial baseline model in accuracy on our benchmarks.

Establishing the competitiveness of neural networks is important as compared to trees, neural networks are way much more flexible in terms of network architectures, the different types of data they can incorporate, and the knowledge transfer between different machine learning tasks. Several previous studies attempted to apply transformers to EHR tasks with pre-training. For example, Li and colleagues developed a transformer-based framework that predicts conditions in future visits (Li et al., 2020). Additionally, Rasmy and colleagues adapted the BERT framework to the EHR domain, pretrained on over 28 million individuals and evaluated two datasets of disease risk prediction (Rasmy et al., 2021). Compared to these studies, our study more explicitly encodes time decay information and tests on a variety of outcomes beyond disease risk, including adverse events, adverse drug effects, readmission, and misdiagnosis. This effort further broadened the application of EHR-deep learning models.

The superiority of LSTM and transformers in other fields like NLP have been established (Kamath et al., 2019; Yildirim and Asgari-Chenaghlu, 2021), and it is surprising that in our evaluation they should be superseded by simpler models built with time decay. We believe that our results show that longitudinal healthcare data is very different in nature from NLP data. We now explain the difference as follows. In NLP, the meaning of a sentence is determined not only by the combination of multiple words, but also by the word order. It is seldom the case that one word or two alone determines the meaning of the sentence. Because the logical relationship between words, as governed by grammar, is complicated, deep neural networks are necessary to capture the latent meaning as conferred by such word relationships. In longitudinal healthcare data, however, the influence of a single crucial condition can be fundamental. Other events surrounding the crucial condition could well be the consequence or corresponding treatment of the condition and are not as important in influencing future events. We evaluated multiple pooling techniques such as average pooling, sum pooling, and the combination of them. The fact that max-pooling works best (better than max +average) means that the strongest single signal in each feature dimension is most important in predicting future events. Our work does not preclude the research into deeper network architectures; it simply shows that bespoken architectures in other fields cannot be directly borrowed. Rather, we believe that either more customization is needed for existing deeper architectures to work with longitudinal healthcare data, or new designs need to be invented. Our experiments also confirm that transfer learning with a large generic background dataset does help training models for specific tasks.

### Limitations of the study

Nevertheless, there remain several directions worth future exploration following this study. First, for each type of evaluation (disease, adverse event, drug response, readmission, misdiagnosis), we only had the opportunity and space to evaluate a couple of tasks, future large-scale, detailed analysis of more data will inform us of the accurate application domains of the method. Second, we revealed from this study the importance of modeling time decay. Yet the modeling of time decay is currently limited to an exponential function; whether there are better modeling functions remains to be explored. Thirdly, we observed a heavy computational burden for training and pre-training the models; processing infrastructure was specifically designed for this study to speed up the process. To have the pretrained model deployed to hundreds or thousands of tasks, further development of deployment infrastructure is needed. Of note, we do not expect perfect prediction by simply adjusting the models, as model performance is impacted by a number of factors related to the accuracy of the data. For example, we found approximately 1.45% of the patients have an event after the death date, which is an indication of data imperfection. Many disease risks and adverse events not only rely on past medical history but also environmental and hereditary factors.

## STAR★Methods

### Key resources table

**Table:** 

REAGENT or RESOURCE	SOURCE	IDENTIFIER
**Software and algorithms**		

OMOP concept embedding	This paper	https://github.com/Merck/OMOP-CONCEPT-EMBEDDING
PLP	Reps et al. (2018)	https://github.com/OHDSI/PatientLevelPrediction

### Resource availability

#### Lead contact

Further information and requests for resources and reagents should be directed to and will be fulfilled by the lead contact, Boshu Ru (boshu.ru@merck.com).

#### Materials availability

No physical materials were developed by or for this project.

### Method details

#### Data and data processing

We used the Optum Clinformatics Data Mart database (henceforth called “Optum dataset”). The database contains administrative health claims for approximately 69 million unique members of large commercial and Medicare Advantage health plans over the period from 2007 to 2020. All data were de-identified and consistent with the Health Insurance Portability and Accountability Act (HIPAA), therefore, patient informed consent and institutional review were not required. The original data was converted into OMOP common data model following the same process published by Janssen (Blacketer, 2021).

#### Patient outcome prediction tasks

We collected the following binary patient outcome prediction tasks, all implemented in the PLP framework. Multiple target cohorts, outcome cohorts and population settings are used following the original study (Table S1).

• EhdenRA (Yang and Williams, 2020): adverse health outcomes amongst adult rheumatoid arthritis (RA) patients initiating treatment of methotrexate monotherapy. The original study defined 11 different adverse outcome cohorts, from which we picked three representative ones: stroke, acute myocardial infarction (acute_MI) and all infections.
• Stroke (Reps et al., 2020): the original publication used PLP to validate existing models for predicting stroke in female patients newly diagnosed with atrial fibrillation. We used the cohort construction protocols and parameters in the original study for our benchmarking purpose. The study defines two target cohorts: all patients (Stroke) and patients of age ≥ 65 (Stroke65). For each target cohort, two population settings are used: unlimited risk window (… -1) and risk window of 365 days (… -2).
• Bipolar (Lambert and Reps, 2020): this is a package for validation of models predicting bipolar disorder misdiagnosed as major depression disorder (MDD).
• Heart (Wang et al., 2021): predicting 30-day (Heart-1) and 90-day (Heart-2) readmissions in hospitalized patients with heart failure with reduced ejection fraction (HFrEF). The cohort index date is the discharge date.

We used the original code of these PLP packages to construct cohorts, and we preserved their original population settings. We set the maximal sample size to 100,000 patients for all studies. For some of the tasks millions of target patients exist in the Optum dataset, and some of the machine learning methods cannot efficiently handle such a large sample size. Limiting the sample size also puts data sizes of various tasks to the same scale. In all studies, one input sample is one individual’s clinical events (represented by OMOP concept IDs) in the observation period and the start dates of these events (actual dates or dates relative to the end of observation period). An example of an individual’s clinical history can be illustrated using a synthetic data here, https://atlas-demo.ohdsi.org/#/profiles/SYNPUF1K/205.

#### Map-reduce-based framework supports fast longitudinal healthcare data preprocessing

The prediction tasks, with sample size limitation, can be comfortably handled by the PLP library. However, Optum is a massive dataset containing longitudinal healthcare data for more than 69 million patients. The data is stored in a Redshift database, and must be converted into a more efficient format that can be used to train deep learning models.

We developed a map-reduce framework for data processing. The data records, after loaded from the database, are first hashed into N (N = 1000) partitions in the mapping phase. This ensures that 1) for any specific patient, all data of that patient goes into only one partition. 2) each partition can be fully loaded into memory for efficient processing. Multiple mapper threads are used to read from the database in parallel to improve throughput. In each partition, however, records from different source database tables are stored unordered. In the reducing phase, the records of each partition are grouped by patients, sorted by time and converted into a uniform format. This phase is also processed in parallel. After processing, the partitions are saved in an efficient binary format and compressed for less storage overhead and efficient loading.

The raw data loaded from Optum dataset, including the timestamp, concept and a few other fields is about 200GB. After processing, the partition files take up 53GB. The process can be finished using a single server within 8 h.

#### Model training

We run patient-level prediction tasks in three phases. First, we run the respective study package to create the cohorts, without actual model training. Second, we use a modified version of EhdenRA PLP code which allows dynamically loading experimental configuration to evaluate the Random Forest model for all studies. The AUC scores are reported, and the training and testing splitting of samples is recorded. Third, the samples and splitting used by PLP are loaded to evaluate the LightGBM and neural network models.

For neural network training with mini-batches, we limited the length of time series to 768 (including the EOS token). We pad short samples with preluding zeros and discard the earliest events of the long samples. We constructed a dictionary of 10,000 most popular OMOP concepts in Optum dataset and discarded all events whose concepts are not in the dictionary (dictionary handling, especially how to make use of the concept hierarchy, can be improved in future work). Figure S1 plots distribution of domain and vocabulary of these concepts. There are cases when the testing sample contains concepts that do not occur, or occur very scarcely in the training set (the dictionary was constructed with a generic background, which does not guarantee the frequent occurrence of a concept in a specific task). The embedding of such rare concepts are essentially untrained and once appear will jeopardize the prediction. We introduced a whitelist mechanism, in which we discard all concepts in the testing set that occur less than a frequency threshold (50) in the training set. We applied random event dropping at training time as data augmentation. We evaluated various dropping rates, and ended up with a dropping rate of 50%. The effectiveness of this high dropping rate also confirms that longitudinal healthcare data-based predictions depend more on the feature contribution of a few crucial events, rather than the hidden logic between all the events.

#### Fine-tuning

The easiest way to use the pretrained models for downstream prediction tasks is to initialize the embedding layers with pretrained weights. However, without making other adjustments, we observed that the networks converge to AUC values slightly less than as if the downstream networks are trained from scratch. The networks do converge substantially faster than from-scratch training, which confirms that the pretrained weights do carry meaningful information. We were able to achieve improvements with the following two-stage fine-tuning procedure. In the first phase, we freeze the embedding layers to pretrained weights, and use regular learning rates to train the rest of the network for 5 epochs. In the second phase, the whole network is trained with 1/10 regular learning rate. These parameters were obtained by manual experiments as systematic grid search is unrealistic with this data size and time of running.

## Data Availability

The data that support the findings of this study are available from Optum but restrictions apply to the availability of these data, which were used under license for the current study, and so are not publicly available. Optum dataset is however available from the authors upon reasonable request and with permission of Optum. Source code is open with GPL license and available at https://github.com/Merck/OMOP-CONCEPT-EMBEDDING.
