# Supplementary material for: Generic medical concept embedding and time decay for diverse patient outcome prediction tasks
Source: iScience. 2022 Aug 4;25(9):104880. doi: 10.1016/j.isci.2022.104880 (PMC9418804; doi:10.1016/j.isci.2022.104880)
Supplement: Document S1. Figures S1–S3 and Tables S1–S5 [file mmc1.pdf]

iScience, Volume *25*

Supplemental information

Generic medical concept embedding and time decay for diverse patient outcome prediction tasks

Yupeng Li, Wei Dong, Boshu Ru, Adam Black, Xinyuan Zhang, and Yuanfang Guan

Figure S1. Distribution of concepts across vocabularies grouped by domain, Related to Figure 1.

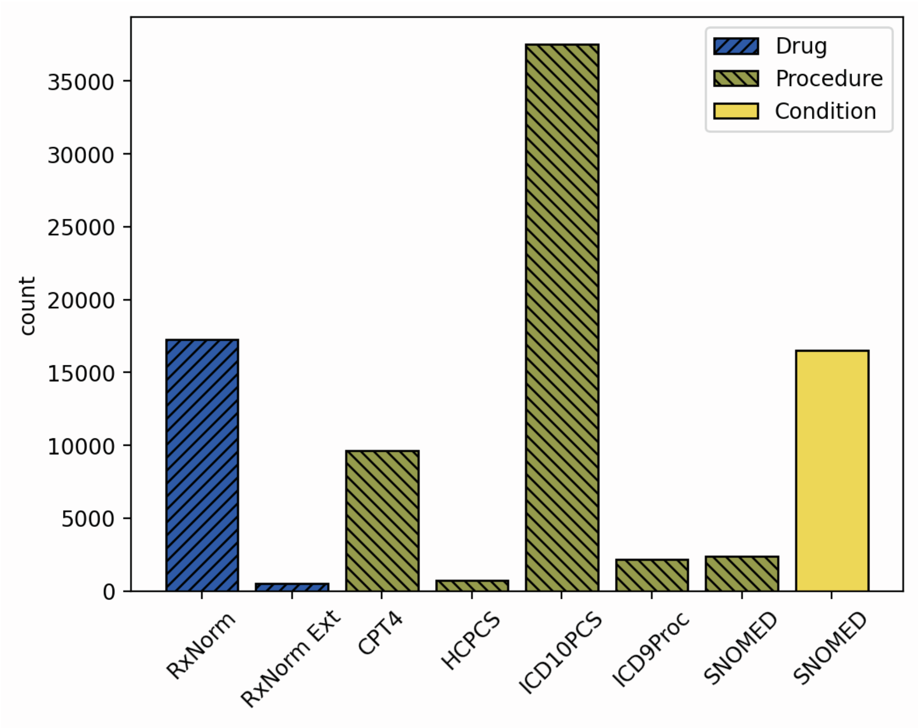

Figure S2. Evaluation of more transformer architectures, Related to Figure 3. Transformer architecture BxD means the network has B blocks of transformers, with an embedding number of dimensions of D. “Trans 4x512” is Google’s BERT-Small architecture. Overall, we observe that 1) transformers are not better than LSTM Max and Baseline (Max). 2) Max is better than EOS in all cases except for 2 settings in RA-acute_MI. Below are the rationales of the B and D values. a. The pretraining logic of BERT class for a deeper architecture, and D=512 is a typical setting for Google’s BERT. And with a deeper network, we are not able to use D=1024 for pretraining due to memory constraints. So we chose 4x512 to be the architecture for a combination of pretraining + finetune. (The figure also shows that for B=4, D=512 is actually slightly better than D=1024.) b. Without pretraining, our evaluation shows that 1x1024 works pretty well and is way more efficient than deeper architectures.

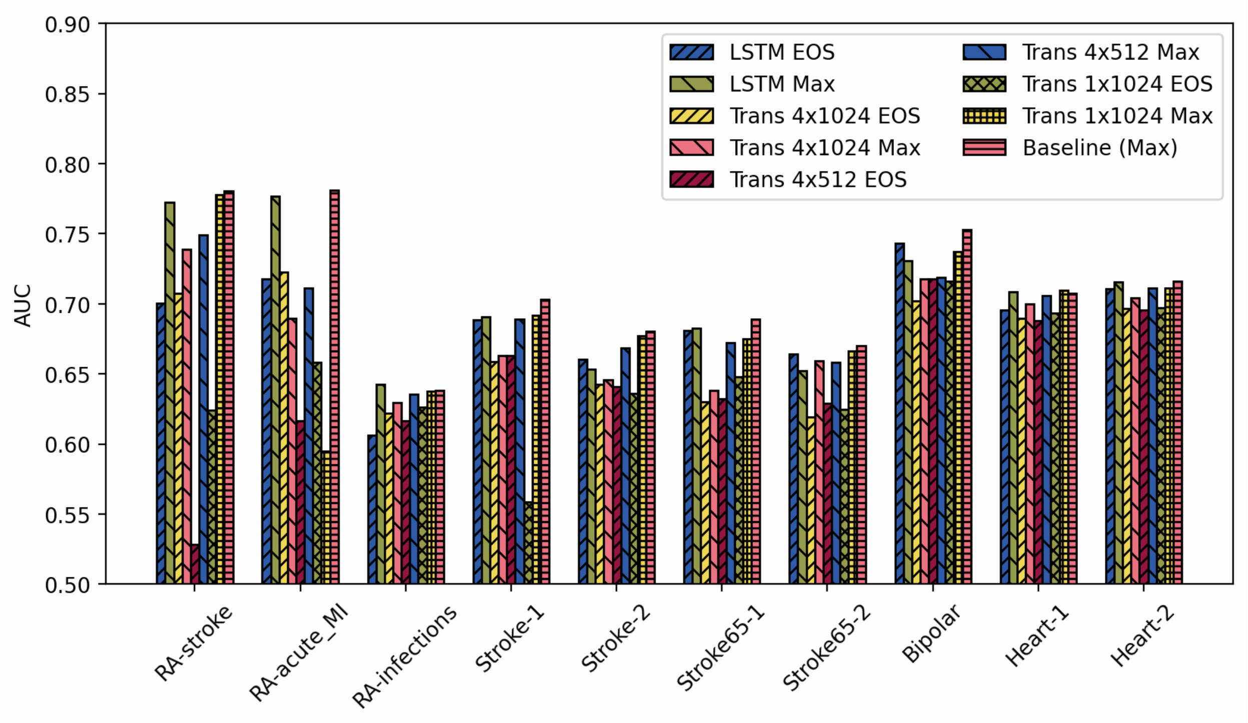

Figure S3. Performance comparison of different pre-training strategies with BERT/MLM (B=4, D=512) added, for the sake of comparison against existing art, Related to Figure 3. Cosine or cross entropy showed consistently better performance.

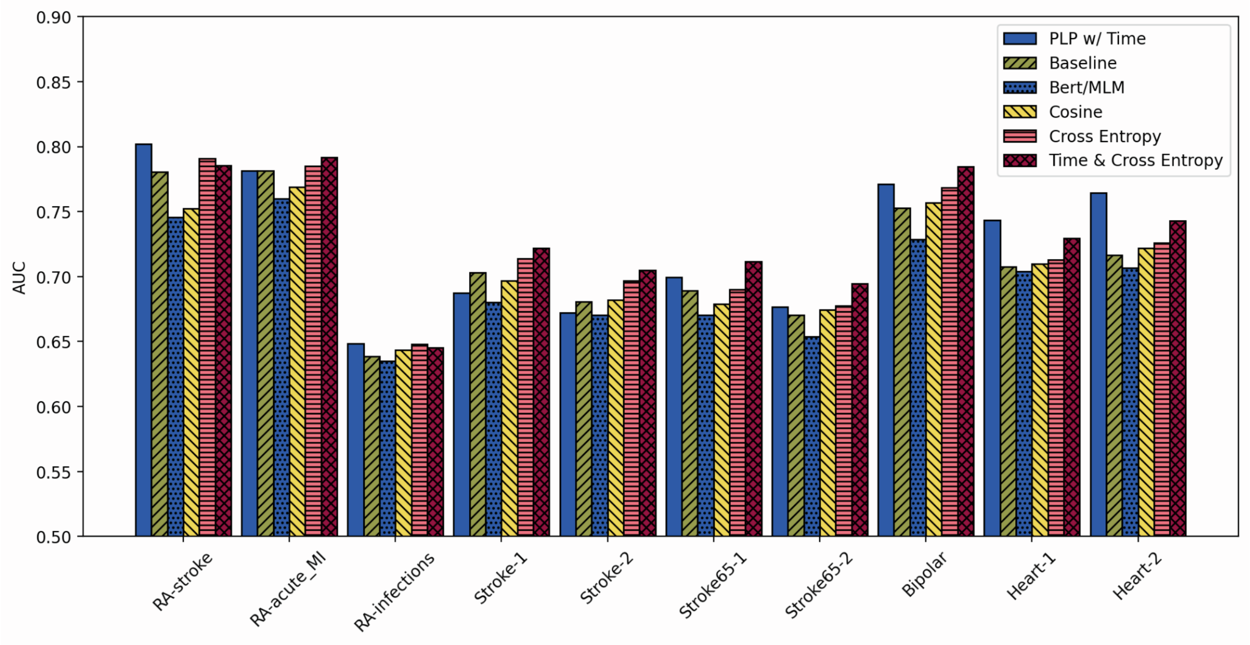

Table S1. Number of examples in training and test for each study, Related to STAR Method Details – Patient Outcome Prediction Tasks.

**Study**	**Negative examples in training**	**Positive examples in training**	**Negative examples in test**	**Positive examples in test**
RA-stroke	12592	334	4203	117
RA-acute_MI	12691	270	4247	78
RA-infections	6591	4942	2189	1656
Stroke-1	36634	2133	9090	548
Stroke-2	53291	2757	13243	687
Stroke65-1	35874	2337	9104	613
Stroke65-2	52640	2963	13125	793
Bipolar	46068	710	15444	240
Heart-1	65399	4942	21813	1571
Heart-2	54089	8190	18090	2613

Table S2. Performance by baseline methods and study, Related to Figure 1C

Method	RA-stroke	RA-acute_MI	RA-infections	Stroke-1	Stroke-2	Stroke 65-1	Stroke 65-2	Bipolar	Heart-1	Heart-2
PLP/ Random Forest	0.798	0.778	0.638	0.685	0.671	0.684	0.672	0.757	0.731	0.751
PLP w/Time	0.802	0.782	0.649	0.687	0.672	0.700	0.677	0.771	0.743	0.764
LightGBM	0.708	0.708	0.620	0.677	0.664	0.640	0.628	0.746	0.739	0.767
Baseline	0.781	0.781	0.638	0.703	0.681	0.689	0.670	0.753	0.707	0.716
Time & Finetune	0.785	0.792	0.645	0.722	0.705	0.712	0.694	0.785	0.730	0.743

Table S3. Performance by alternative neural network architectures and study, Related to Figure 2B

Method	RA-stroke	RA-acute_MI	RA-infections	Stroke-1	Stroke-2	Stroke 65-1	Stroke 65-2	Bipolar	Heart-1	Heart-2
LSTM EOS	0.701	0.718	0.606	0.688	0.660	0.681	0.664	0.743	0.696	0.711
LSTM Max	0.773	0.777	0.643	0.691	0.654	0.682	0.652	0.730	0.708	0.716
Transformer EOS	0.624	0.658	0.626	0.559	0.636	0.648	0.625	0.716	0.693	0.697
Transformer Max	0.778	0.595	0.638	0.692	0.677	0.675	0.666	0.737	0.709	0.711
Baseline (Max)	0.781	0.781	0.638	0.703	0.681	0.689	0.670	0.753	0.707	0.716

Table S4. Performance of baseline and time decay models by study, Related to Figure 3C

Method	RA-stroke	RA-acute_MI	RA-infections	Stroke-1	Stroke-2	Stroke 65-1	Stroke 65-2	Bipolar	Heart-1	Heart-2
Baseline	0.781	0.781	0.638	0.703	0.681	0.689	0.670	0.753	0.707	0.716
Time Encode	0.770	0.776	0.651	0.696	0.677	0.680	0.667	0.750	0.714	0.723
Time Decay	0.773	0.776	0.642	0.708	0.689	0.704	0.686	0.772	0.729	0.737

Table S5. Performance of by pre-training strategies and study, Related to Figure 4C

**Method**	**RA-stroke**	**RA-acute_MI**	**RA-infections**	**Stroke-1**	**Stroke-2**	**Stroke 65-1**	**Stroke 65-2**	**Bipolar**	**Heart-1**	**Heart-2**
PLP w/ Time	0.802	0.782	0.649	0.687	0.672	0.700	0.677	0.771	0.743	0.764
Baseline	0.781	0.781	0.638	0.703	0.681	0.689	0.670	0.753	0.707	0.716
Cosine	0.752	0.769	0.644	0.697	0.682	0.679	0.675	0.757	0.710	0.722
Cross Entropy	0.791	0.785	0.648	0.714	0.697	0.690	0.678	0.769	0.713	0.726
Time & Cross Entropy	0.785	0.792	0.645	0.722	0.705	0.712	0.694	0.785	0.730	0.743
